# Self-reproduction as an autonomous process of growth and reorganization in fully abiotic, artificial and synthetic cells

**DOI:** 10.1073/pnas.2412514122

**Published:** 2025-05-27

**Authors:** Sai Krishna Katla, Chenyu Lin, Juan Pérez-Mercader

**Affiliations:** ^a^Department of Earth and Planetary Sciences, Harvard University, Cambridge, MA 02138-1204; ^b^Santa Fe Institute, Santa Fe, NM 87501

**Keywords:** polymerization-induced self-assembly, protocells, self-reproduction, artificial life, origin of life

## Abstract

We investigate mechanisms for the observed nonlinear growth in the number of polymer vesicles generated during a photo-Reversible Addition-Fragmentation Chain Transfer-based polymerization-induced self-assembly (PISA) reaction. Our experimental results reveal the presence of a self-reproduction process during which chemically active polymer protocells are chemically and autonomously generated in a light-stimulated one-pot reaction that starts from a homogeneous blend of non-self-assembling molecules and which, as observed microscopically, form vesicular objects that grow and multiply (reproduce) during irradiation with green light (530 nm) as the reaction proceeds. By using a filtration-based protocol, our experiments demonstrate that the self-reproduction process occurs concomitantly with the PISA process and results in a nonlinear increase in the number of polymer vesicles during photopolymerization which can only be ascribed to their reproduction via polymeric spores ejected from previously existing first-generation vesicles. The second and subsequent generations’ vesicles also self-reproduce and continue the process of population growth.

## Significance

Self-reproduction is one of the most fundamental features of natural life. This study introduces a biochemistry-free method for creating self-reproducing polymeric vesicles. In this process, nonamphiphilic molecules are mixed and illuminated with green light, initiating polymerization into amphiphiles that self-assemble into vesicles. These vesicles evolve through feedback between polymerization, degradation, and chemiosmotic gradients, resulting in self-reproduction. As vesicles grow, they polymerize their contents, leading to their partial release and their reproduction into new vesicles, exhibiting a loose form of heritable variation. This process mimics key aspects of living systems, offering a path for developing a broad class of abiotic, life-like systems.

Among the characteristics shared by all extant living systems (1–3), their chemically controlled self-reproduction ranks perhaps as one of the most spectacular and exclusive. Under the control of their internal chemical networks, and in tight relationship with their environment from where they harvest food, energy, and information, natural living systems spawn representations of themselves (reproductions) as fully functional systems which also autonomously and under sufficiently similar conditions to those of their progenitors are again capable of self-reproduction into new generations. Indeed, when living systems reproduce, their species continues to exist via their progeny and propagates as such into the future while also enabling fundamental aspects of their Darwinian evolution due to the generation by reproduction of populations which include heritable variation (4).

In extant, biochemistry-based life, even for the simpler single-celled living systems such as bacteria, the process of reproduction is complex and involves many complicated and coordinated steps. These take place within the cell under the precise control of biochemistry and collectively constitute the cell division cycle (CDC) (5). Like Virchow said in 1858 “omnes cellula e cellula” or “every cell comes from a pre-existent cell” (6–8).

However, we may ask: Is extant biochemistry (9) with its delicate and well-tuned complexity over billions of years of evolution on Earth necessary for a protocellular (10) chemical system to self-reproduce? Can one construct in the laboratory, nonbiochemical, compartmentalized chemical systems capable of autonomous self-assembly and self-reproduction? Answering these basic questions is important for systems chemistry, artificial life, for protocell research seeking to understand what life may have been like before the advent of biochemistry or Last Universal Common Ancestor (LUCA) during a so-called protocell era and for understanding the potential for life in the Universe, including exoplanets (11–13). During the protocell era, “life must have been simpler” (10, 12) and hundreds of millions of years could have elapsed evolving the transition from chemistry to some form of generalized life (14, 15), which eventually lead to LUCA. Finally, these questions are also central in the old, but nascent, field of synthetic artificial life based on nonbiochemical material implementations of fully synthetic chemical systems capable of mimicking natural living systems (10, 11, 13, 16–20).

In an effort to explore the answer to the above broad questions about the necessity of biochemistry, perhaps helping to trace back life’s origin to the “combination of carbon chemistry and the physics of self-organization,” (21, 22) and building upon the opportunities in precisely this direction offered by polymerization-induced self-assembly (PISA) (20, 23–29), we have designed a PISA batch reactor where in an aqueous solution and strictly avoiding any biochemical molecules, we synthesize amphiphiles that self-organize, self-assemble, and self-boot into chemically active micelles. (In this paper the term boot is used in the sense of computer science.) The micelles then develop into functional giant vesicular systems (GV) from just a few small (and low complexity) molecular species chosen ad hoc by us for implementing PISA. Starting from a homogeneous blend of several such simple molecules, our systems exclusively rely on chemical forces controlling the exchange of atoms among molecules, together with their physicochemical implications (30), and use light as a source of external energy. The aqueous thermostated and illuminated reaction mixture contains a hydrophilic polymer with a small, and slightly hydrophobic chain transfer agent molecule (CTA) bonded at its end (this can be further simplified if desired, cf. refs. 31 and 32), plus some selected monomers such as acrylonitrile or hydroxypropyl methacrylate (HPMA) that can be photopolymerized to the above hydrophilic polymer to yield amphiphilic block copolymer molecules. Finally, the PISA mixture also contains at least one photocatalyst or iniferter (a photocatalyst molecule that acts as *ini*tiator, chain transfer agent, and *ter*minator of the polymerization reaction) (33) for the photopolymerization reaction. [Note that this very simplified out-of-equilibrium system can abstractly be (34) imagined as a laboratory version of a “warm little pond” where the notion of heterotrophic abiogenesis can be explored.]

After illumination is switched on, the chemical reactions start and the initially homogeneous blend produces amphiphile molecules in the bulk. As the concentration of this amphiphile increases beyond its Critical Micelle Concentration (CMC), the activity of the chemical reactions leads to liquid phase separation due to the ongoing polymerization process of the hydrophobic block in the amphiphiles, which eventually self-assemble and self-organize into micelles (35). During their self-assembly in the course of the living polymerization synthesis of the amphiphiles, these micelles entrap some of the current reaction medium, which guarantees the continuation of the polymerization reaction in their interior, although at a rate different from the one in the bulk which, consequently, generates and amplifies osmotic gradients (19, 36). In other words, by simultaneously changing the packing parameter value (30) of the living [in the sense of living polymerization (37)] amphiphile due to the increase in the length of its hydrophobic block, the ongoing internal photopolymerization reaction also powers the dynamical morphological evolution of the micelles which develop in time and eventually become micron scale vesicles (loosely called GVs). The crucial chemical difference between the materials and conditions inside the membrane and within the vesicle on the one hand, and on the other hand the bulk (environment) within which the vesicles booted-up and now exist, gives rise to a series of events which (for appropriate PISA formulations) manifest in the course of the optical microscopy observation of the giant vesicles undergoing this process.

Prominently among the above events is that during the photopolymerization (38) reaction under microscope illumination, a striking nonlinear increase in the number of self-assembled vesicular structures (19, 34, 39, 40) takes place. This immediately leads one to ask whether a) the observed increase in vesicle number is caused only by the inherent production of newer vesicles that once were smaller nano-scale micelles that developed into vesicles which are growing due to photopolymerization and consumption of chemicals during the polymerization reaction or b) if, in addition to the above, there is a contribution due to the self-reproduction of older vesicles that had previously been generated during the ongoing PISA process. We will study in this paper the details of this nonlinear increase in the number of vesicles during PISA and find b) to be the case.

## Results and Discussion

To study this increase and its causes, we first examined the chemistry of the system and the conditions under which the polymerization reaction takes place. In our experiments, we synthesized amphiphiles that eventually self-assembled as polymer micelles which then grew into vesicles in water using a Reversible Addition-Fragmentation Chain Transfer (RAFT) photopolymerization procedure with 4-Cyano-4-[(dodecylsulfanylthiocarbonyl)sulfanyl]pentanoic acid (CDTPA) as the CTA *SI Appendix*, Fig. S1. The photoreactor for the synthesis consisted of 45 green Light Emitting Diode (LED)s (λ = 523 nm; single LED power = 2.97 mW) circularly wound around the outside surface of a 100 mL glass beaker inside which we placed a capped 10 mL glass reaction vial (cf. *SI Appendix*, Fig. S2).

The reaction vial contained an aqueous solution of a macro-RAFT agent with 32 units of ethylene glycol bonded to the above CDTPA molecule (PEG_32_-CDTPA), plus the monomer HPMA to be polymerized to the macro-RAFT agent and build in an inert atmosphere (vial filled with N_2_) the hydrophobic chain of an amphiphilic block copolymer. The photopolymerization reaction was carried out for 90 min at 33° C, *SI Appendix*, Fig. S2, while illuminated by the LEDs. The degree of polymerization or length of the poly-HPMA block in the amphiphile was characterized using NMR to be 104 (41), *SI Appendix*, Fig. S3. The resulting self-assembled structures eventually generated by the self-organization and self-assembly processes associated to the photo-RAFT PISA were characterized using Scanning Electron Microscopy and Transmission Electron Microscopy as spherical vesicles with average diameter between 1 and 2 µm, *SI Appendix*, Fig. S4. To accelerate the growth in vesicle size during polymerization, we added to the initial blend Zinc tetraphenylporphyrin (ZnTPP) as a photocatalyst that has previously been reported to work in PISA photopolymerization reactions (38).

A small aliquot (50 μL) of this solution was then transferred to a standard microscope glass slide provided with a square frame (25 mm × 25 mm) which was then sealed by a glass coverslip. These vesicles were then observed using the green light of the optical microscope to quantify and analyze the increase over time in their number (population) as recorded in time-lapsed images captured with the optical microscope’s digital camera, *SI Appendix*, Fig. S5.

Once transferred to the microscope slide, our chemical system continues to be thermodynamically open and out of equilibrium since it is subject to the effects of light and temperature in its environment. (But it also remained closed to the feed of external chemical reagents.) It is important to bear in mind for what follows that just as in any one-pot (batch) RAFT-PISA reactor, the HPMA monomer in the microscope slide continues being irreversibly consumed and leading to the formation of more amphiphiles, which then self-organize and self-assemble into micelles which grow in size to accommodate the increasing length of the hydrophobic block of the amphiphiles constituting the already self-assembled objects. In our experiments, we observed that during the course of the PISA process, the average size of the vesicles did not increase monotonically but reached a maximum size which then decreased over time.

Furthermore, the above decrease in average size came accompanied by a rapid (and clearly nonlinear) increase in the number of vesicles which cannot be attributed only to the newer vesicles produced from the polymerization process occurring through conventional PISA (cf. Fig. 3). And since after transfer of the PISA reaction aliquot to the microscope slide no additional ingredients were added to the chemically developing system within the slide, we conclude that without some amphiphile ejection from the interior of the existing vesicles somehow taking place, the number of vesicles will not increase only due to direct polymerization.

**Fig. 1.**
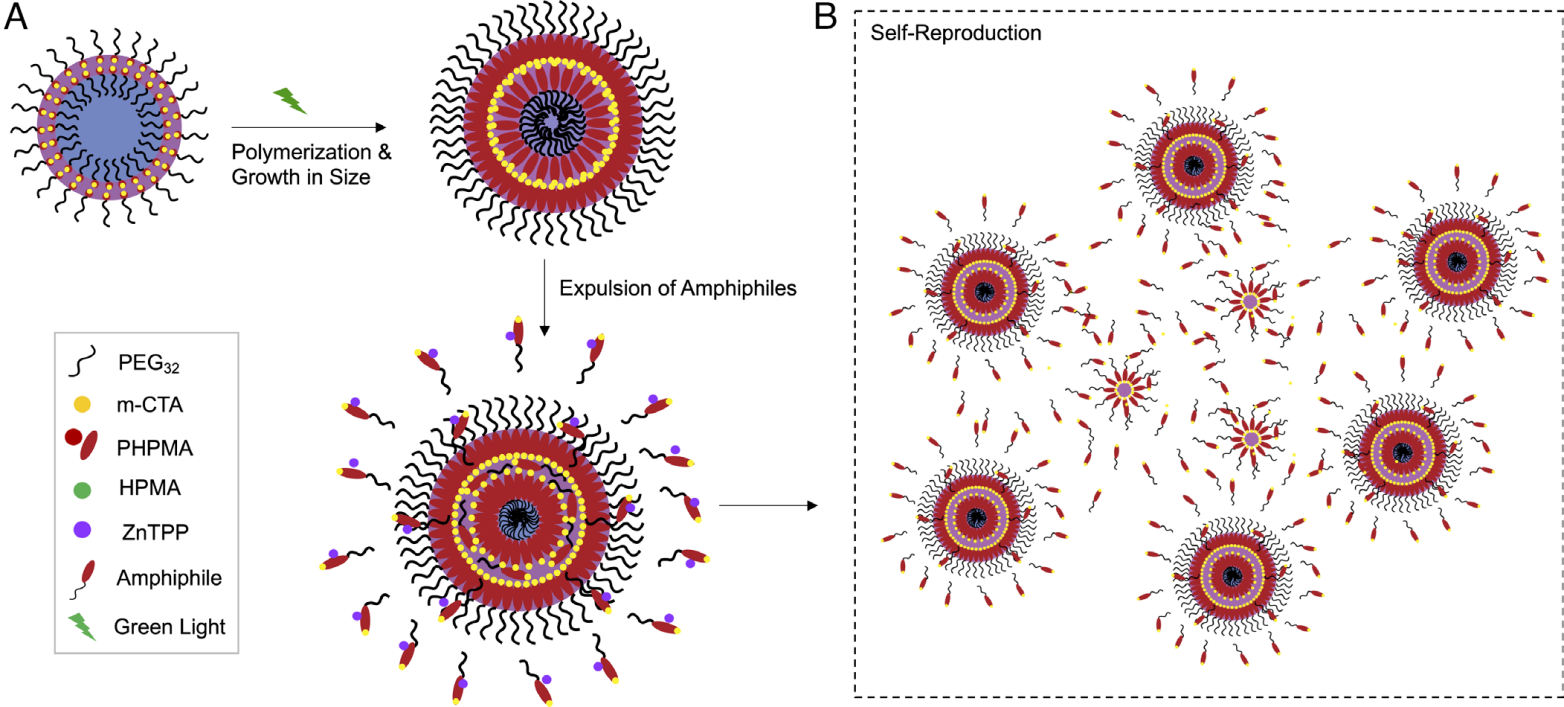
(*A*) Illustration showing the different stages of polymer vesicle growth leading to the action of expulsion of amphiphiles. (*B*) Illustration showing the formation of new vesicles from the reorganization through self-reproduction of amphiphiles expelled into the bulk.

In order to identify which causes may underlie the observed nonlinear growth in vesicle number, we investigated in detail the steps involved in the photopolymerization process and focused on two major changes occurring in the system as the polymerization progresses. Because of the interaction of ZnTPP with green light, *SI Appendix*, Fig. S6, faster polymerization occurs and leads to the extension of the hydrophobic block of amphiphiles already within vesicles. As this intravesicle polymerization progresses, the vesicle membrane grows thicker (due to the increasing length of the hydrophobic block). This creates an osmotic imbalance with respect to the medium and amphiphile congestion in the lumen, which is eventually relieved by ejecting (squeezing) through the membrane (perhaps through the softer or defective parts of the membrane where the Young’s modulus is lower, see *SI Appendix*, Fig. S7) a fraction of the amphiphiles (preferentially those with a shorter hydrophobic block) from inside the vesicle membrane and out into the bulk where the concentration of shorter hydrophobic chain amphiphiles is lower (Fig. 1*A*). Thus, these immature amphiphiles squeezed out of the original (parent) vesicle(s) disperse into its vicinity. There they mix with other partially polymerized amphiphiles and polymerization reaction components locally present in this region of the bulk medium. Provided there are enough monomers around, the living CTA molecule at the end of the hydrophobic tail of the just expelled amphiphiles can still engage in chain elongation together with other partially reacted (with a smaller PI for their hydrophobic part) amphiphiles. The net effect of these processes occurring in the bulk medium is an increase in the local amphiphile concentration which eventually will reach their CMC, self-organize and self-assemble into a new generation of dynamically evolving micelles. These micelles will follow the same fate and dynamically develop into new (younger) vesicles just as did their parent vesicles, and so long as there is food and light around. Note that the partially reacted amphiphiles squeezed out of a vesicle at this stage may not be monodisperse in the chain length of their hydrophobic tails and, therefore, we expect that there will necessarily be some variation in the length of their living hydrophobic blocks. This physical variation provides the basis for a mechanism of loose heritable variation in the next generation of micelles and their resulting mature vesicles with respect to their parents (16, 41, 42). (This form of loose heritable variation arises from the joint effects of the nonlinearity of the RAFT polymerization process, the chemical physics of the self-assembly and polymerization of the chemical spores, and the polydispersity in their partially elongated chain lengths.)

The above process constitutes a form of self-reproduction (Fig. 1*B*) as the newer vesicles are generated from the additional processing and reorganization of shorter amphiphiles ejected (squeezed out) from older and growing vesicles. The cycle of events just described will continue for as long as there is enough food and energy (i.e., HPMA monomer, macro chain transfer agent molecules, and green light) available for the reactions going on in the microscope slide. Put concisely, the PISA-driven amphiphile congestion caused by hydrophobic block extension eventually leads to an increase in vesicle number in the bulk solution and to some variation in the size of the hydrophobic block.

We validated this mechanism of self-reproduction of the polymer vesicles by carrying out a series of ad hoc experiments complementary to the above observations. These were designed to a) separate the contribution to the increase in the number of vesicles due to the ongoing PISA process (direct polymerization) from the proper self-reproduction contribution, b) understand and document the formation of newer (i.e., younger) vesicles by reorganization of amphiphiles from preexisting vesicles and c) to confirm the transfer of ZnTPP molecules from the first generation to the next generation of vesicles.

### Contributions from the Ongoing PISA Process in the Bulk Solution.

**Fig. 2.**
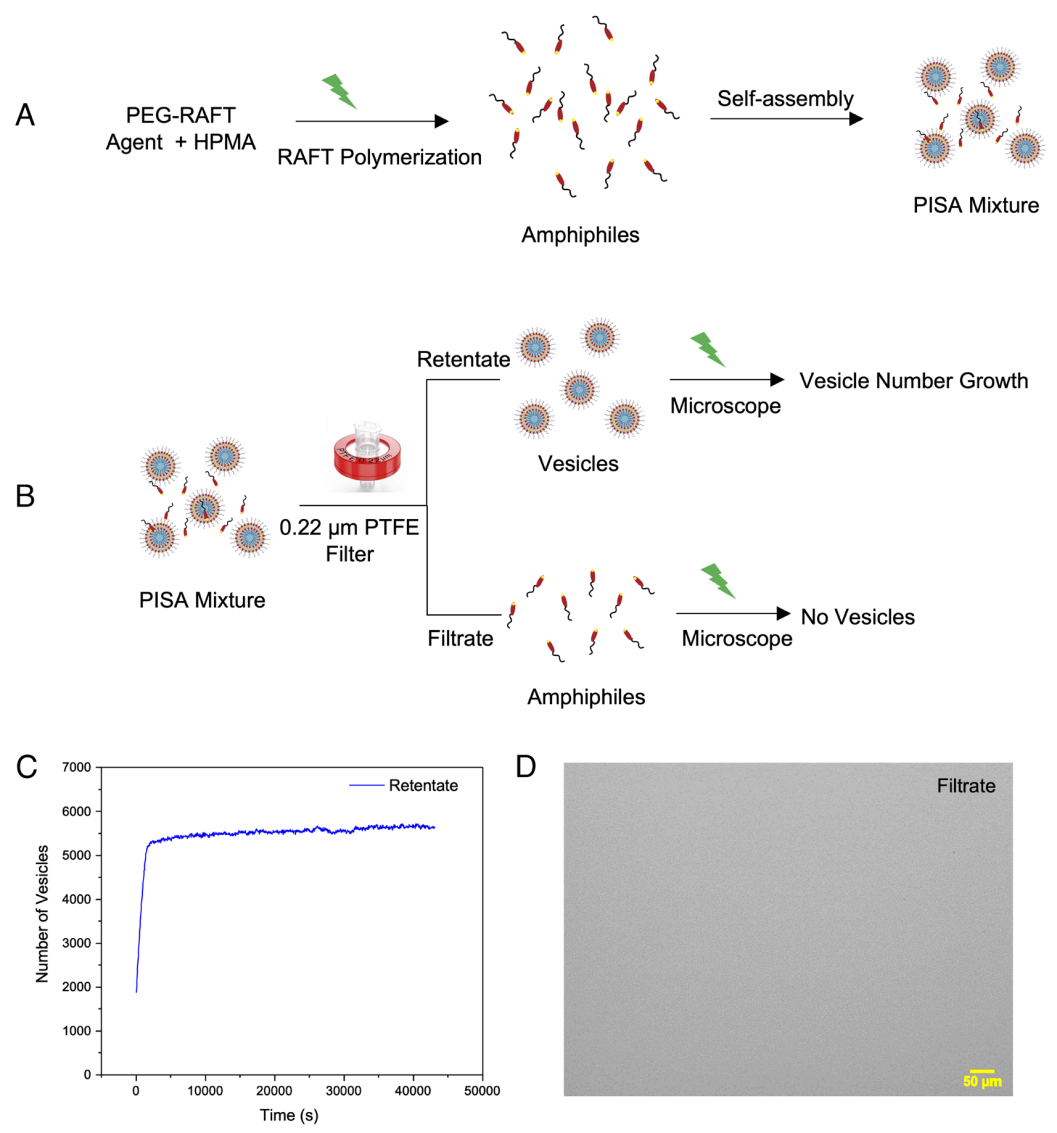
(*A*) The synthesis of amphiphiles and polymer vesicles using RAFT polymerization. (*B*) Filtration of PISA mixture and subsequent irradiation of amphiphiles and vesicles separately. (*C*) Plot showing the growth in the number of vesicles during irradiation of retentate with green light. (*D*) Optical image of the filtrate sample irradiated with green light showing no visible objects in the imaging frame.

**Fig. 3.**
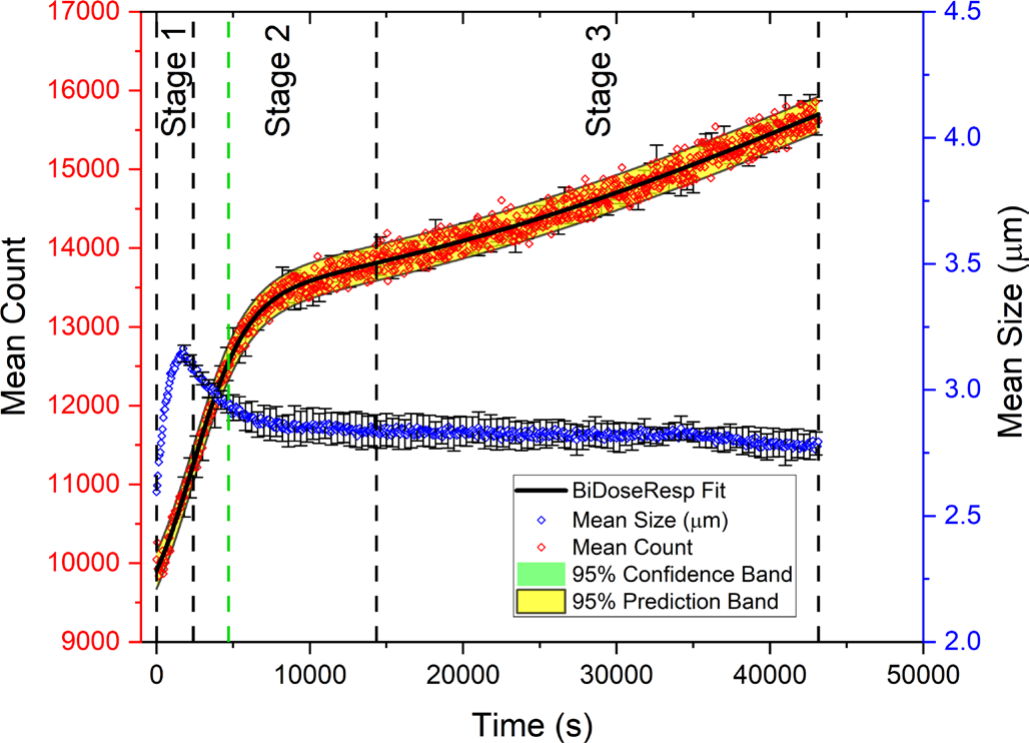
Plot showing the number of vesicles produced over time (red data points) and their average diameter (blue data points) as the PISA system is irradiated with the green light of our microscope. The time coordinate is the time elapsed since the start of the experiments on the microscope. Each data point is the unweighted average of the population and mean diameter measured from three experiments carried out on different dates using our system and the same stock solutions. The error bars are the SD of the Mean (SDOM). As discussed below (*SI Appendix*, Fig. S11), there are three distinct stages in the time evolution of the population of these vesicles determined by analyzing the growth rates for the number of objects (population sizes) and their dynamics. (Note that these vesicles contain ZnTPP molecules which catalyze the photopolymerization reaction.)

The ongoing PISA process contributes to the increase in vesicle numbers (population) since the polymerization taking place within our PISA system produces amphiphiles continuously until the initial amount of HPMA monomer in the system is depleted. Furthermore, as was already discussed, these amphiphiles remain in the bulk and increase their concentrations beyond the CMC at which point they reorganize into micelles (Fig. 2*A*) that could eventually grow bigger (or merge) to become vesicles as the polymerization progresses. In order to determine their contribution to the total vesicle population, we needed to isolate the objects produced by the RAFT polymerization in the PISA process that were observed by optical microscopy (Fig. 3). To implement this, the active PISA solution was filtered through a 0.22 μm PTFE membrane filter to separate already formed vesicles from the unassembled amphiphiles in the bulk medium (Fig. 2*B* and *SI Appendix*, Fig. S8). The retentate only contained the vesicles (*SI Appendix*, Fig. S9*B*), and these increased in number upon photopolymerization through irradiation under the microscope with green light for 12 h (following a pulsed illumination pattern with pulse duration = 1 s and a period between pulses = 10 s) (Fig. 2*C*). On the other hand, when the filtrate (*SI Appendix*, Figs. S8 and S9*A*), which contained the amphiphiles and small micelles, was irradiated under the microscope using the same conditions, no vesicle formation was observed over time (Fig. 2*D*). Since the new vesicles were formed from the retentate vesicles and not from the filtrate, i.e. the amphiphiles left in the bulk, these observations confirm that formation of “newer” vesicles can be attributed to “self-reproduction” from the preexisting vesicles and not through the self-assembly of amphiphiles coming from the ongoing PISA process in the bulk.

### Formation of Newer (Younger Generation) of Vesicles from Preexisting Vesicles.

As the RAFT-polymerization progresses, vesicles continue to be formed in our PISA system. Fig. 3 red trace shows the increase in the population of ZnTPP-containing vesicles as they were irradiated with green light. The generation of new vesicles occurred through the self-assembly of the expelled amphiphiles that are in the vicinity of larger vesicles, a process that can be confirmed by analyzing the time development of the average size of the vesicles in the system, blue trace in Fig. 3, and the size of their population in the imaging area while they were being irradiated with green light from the microscope. One expects the average size of vesicles to increase gradually when the length of the amphiphiles in their membrane increases as the monomer contained inside these vesicles is consumed. In our experiments after an initial synchronized increase in number of vesicles and their average size, the trend reverses, and the average size of the vesicles decreases over time (Fig. 3, blue trace) which is consistent with an increase in their population as the retentate system was being irradiated with green light. This indicates that during the course of this reversed trend, newer, smaller-sized objects are formed in the system from the retentate. Hence, the newly formed vesicles are smaller than their previous generation. But, eventually, the initial trend of decrease in average diameter stabilized and remained constant or slightly decreasing at the same time that the total vesicle population increased.

In other words, after the initial burst of growth seen in the red trace in Fig. 3, the vesicles exhibited significant reduction of their average size, which suggests that newly formed small objects contribute to the reduction of the average global size. This can be attributed to fast polymerization at the beginning of the reaction due to the presence of abundant HPMA which quickly generates newer objects (micelles). With continued polymerization, larger vesicles eject partially polymerized amphiphiles which when reaching appropriate concentrations in the bulk will form new objects, and these new smaller objects also grow up to become vesicles of a certain critical size before they start to eject (squeeze out) partially reacted amphiphiles from their lumens into their vicinity and are then reprocessed to form new self-assembled structures. This, of course, is the quintessential signature of a time-dependent exponential process, and, hence, the observed combinations of exponential growth in the vesicle number (cf. the red-yellow swath in Fig. 3). In addition, as shown in *SI Appendix*, Fig. S13, we also find that a time sequence of histograms of the number of visible vesicles produced as a function of their sizes shows a multimodal size distribution whose number of peaks and their heights change (and grow) with time, as is expected for a dynamically reproducing population. This population dynamics occurs as its size increases at a slow but exponential rate and the average diameter of the population stays roughly constant.

We can now summarize these findings. First, the larger vesicles eject amphiphiles which eventually produce younger small objects (reducing the global average size). Second, when the larger vesicles slow down the ejection of their partially formed amphiphiles, but the smaller objects continue to grow, the average size also remains relatively constant as the population increases. The transition from growth in number but decrease in size can then be directly correlated with and attributed to the depletion of available HPMA monomer in the observed area and consequently with a reduction in the rate of polymerization. In fact, for a developing vesicle, increasing lumen congestion (39) requires sufficient chain extension. Older or larger vesicles, which have built up sufficient congestion in their lumens can eject the amphiphiles (and reduce their overall internal surplus concentration) by consuming a little more food (monomer), which increases the congestion in the membrane. However, the younger and smaller vesicles need more food to first grow and then eject amphiphiles to produce newer small vesicles. Thus, at the beginning, when food is abundant, growth and reproduction are efficient. When food becomes scarcer, it slows down the amphiphile ejection from the larger vesicles and with it the corresponding growth rate of the larger-sized population. In addition, the small younger vesicles can only grow slowly and not eject amphiphiles for some time until they reach a certain size. Putting this together tells us that the net growth in number is delayed while the global average size decreases due to the incorporation of newly reproducing vesicles into the population.

### Transfer of ZnTPP Molecules from First-Generation Vesicles to the Next Generation.

**Fig. 4.**
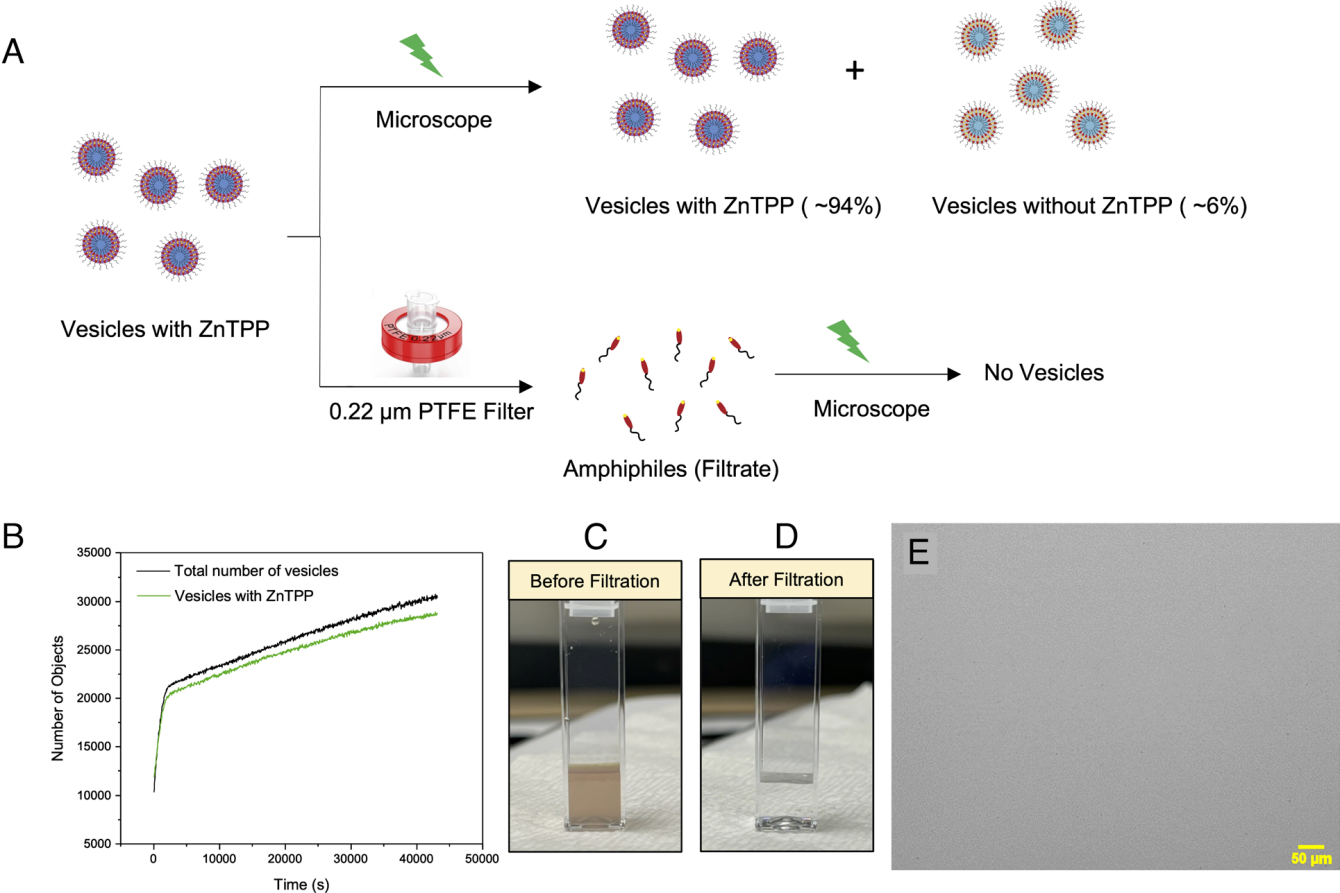
The transfer of ZnTPP molecules from first generation vesicles to next generation. (*A*) Graphical scheme illustrating our filtration experiments, and where we find that ZnTPP stays in the hydrophobic membrane of the population of vesicles (contained in the retentate of the filtering experiment) when it is filtrated through a 0.220 μm PTFE (Teflon) filter. (*B*) Fluorescence imaging of the vesicles with ZnTPP irradiated in green light and showing the percentage of vesicles produced with ZnTPP and without ZnTPP, respectively. (*C*) Filtration experiment. Cuvette showing solution before filtration. (*D*) Filtration experiment. Cuvette showing solution after filtration. (*E*) Microscope image of the filtrate irradiated with the microscope green light and showing that no vesicle formation took place in the filtrate.

In addition to confirming that the newer vesicles are formed through a self-reproduction process, it is also important to check whether the ZnTPP photocatalyst, which is present within the membrane of these growing vesicles, is passed from one generation of vesicles to the next generation. When the vesicles were irradiated with green light, fluorescence imaging showed 94% of the newly formed vesicles contained ZnTPP (Fig. 4 *A* and *B*). Since the amount of ZnTPP photocatalyst in the system is fixed, this confirms that the ZnTPP molecules are passed from the first-generation vesicles down to the second generation during the ongoing self-reproduction process. Taking into account the hydrophobic nature of the TPP macrocycle (in the ZnTPP molecules) and their affinity toward the hydrophobic core-forming block of the amphiphiles leads one to expect that, when the amphiphiles are squeezed out and expelled, the ZnTPP molecules will tend to go with them into the newly formed objects, be sequestered within and become part of their internal contents. To confirm this, we performed another filtration experiment and observed that when the vesicles containing ZnTPP (Fig. 4*C*) were filtered through a 0.22 μm PTFE membrane filter, the filtrate did not show the color of the ZnTPP (Fig. 4*D*) and also that upon irradiation (with the microscope’s green light) of the filtrate, no vesicle formation was observed (Fig. 4*E*). This negative result confirms that the ZnTPP stays within the hydrophobic membrane of the vesicles (in the retentate) during the filtration process. *SI Appendix*, Fig. S12 shows the absorption spectroscopy of the ZnTPP-added vesicles before and after filtration, confirming the absence of ZnTPP in the filtered sample.

### Analysis and Description of the Time Evolution of the Vesicle Population Size.

The population of vesicles produced in our PISA experiments shows three distinct growth regimes, as can be inferred by plotting the number of vesicles vs. time, and is shown by the red-yellow swath around the data points for three independent experiments in Fig. 3. Overall, the data on this swath contain a pattern consisting of three consecutive and clearly differentiated stages in the evolution of the population dynamics. To analyze each of these stages, we can examine the growth rates of the number of objects during each individual stage. These rates can then be used to infer a chemical rationale for the dominant chemical factors underlying each of the stages.

The growth rates characterizing the three stages can be identified numerically by calculating the first and second derivatives with respect to time of the fitted data for the population size curve in Fig. 3. They are displayed in *SI Appendix*, Fig. S11. These three distinct regimes can be readily understood chemically on the basis of HPMA monomer consumption in the illuminated area over the microscope slide on which the observations for Fig. 3 were made (which was 0.7 mm^2^ or 0.4% of the unilluminated area). Stages 1, 2, and 3 correspond to the three different time-slices we selected in Fig. 3. Stage 1 includes time from the initiation of the observation (t = 0) to approximately 2,400 s into the time-lapsed imaging observations, Stage 2 times between 2,400 s and around 14,350 s, and Stage 3 times between 14,350 and 43,150 s when we ended the observations.

During Stage 1, the HPMA concentration in the slide is high, polymerization starts, is very rapid and is accompanied by a high growth rate of vesicle formation. This fast growth of the vesicle population consumes and depletes the locally available amount of HPMA monomer in the illuminated (by green light) area of the microscope slide, and by about 2,400 s into the reaction, the rate of population increase had reached the limit and a very obvious deceleration leading to a new stage of vesicle population dynamics, which we call Stage 2. This new stage starts with the just mentioned ongoing deceleration but, contrary to what one would expect for a system approaching equilibrium, it does not coast down, and at around 4,700 s slowly and almost unnoticeably accelerates for a long period of time. For the formulation of our reactions, this allows for the previously HPMA-depleted areas in the field of view to recharge by the diffusion of the HPMA present now in a higher concentration in the area surrounding the illuminated region where the HPMA food was depleted during Stage 1. At about 14,350 s, the rate of vesicle production starts to increase again and enters a new regime we call Stage 3 during which HPMA consumption accelerates, although at a much slower rate than in Stage 1 and the newly produced vesicles start contributing to the population. Finally, after reaching a maximum at about 30,000 s, the growth rate begins to decelerate again with a very slow convex upward trend which reminds one of a sigmoidal growth (43).

## Conclusions

Reproduction is among the most fundamental functions in natural living systems. It can assure the survival and continuity of a species and can enable some of biology’s most characteristic features, such as species population behaviors and, when it includes heritable variation, Darwinian evolutionary phenomena (4). Presumably, self-reproduction has been present from early in the history of life on Earth and today, employing biochemistry as the chemistry of extant life, is at the core of the universal CDC (5). In the simplest forms of extant life (e.g., in most bacteria), reproduction proceeds through a type of self-reproduction in which a living system on its own and under the control of its biochemistry, generates at least one additional fully functional instance of itself.

We have asked ourselves whether biochemistry and its attendant complexity are necessary for the self-reproduction that we observe in natural (extant) living systems or whether it could also happen in our fully synthetic, strictly nonbiochemical, and far simpler polymeric systems. We find that biochemistry is not necessary, but of course is sufficient.

In this paper, we present experimental results of a detailed study of system self-reproduction in emergent nonbiochemical polymeric vesicular populations created with PISA. These vesicles emerge autopoietically from an initial heterotrophic and homogeneous mixture of (nonbiochemical) molecular species which undergo a RAFT (26) photopolymerization reaction in a liquid medium (H_2_O in this study) that transforms the initial molecules into amphiphilic block copolymers. After the reaction starts, when the CMC for the emerging synthesized amphiphiles in the medium is reached, the amphiphiles autonomously self-organize and self-assemble into functional micelles which entrap the original reaction and under its control further develop and transform into vesicles. A graphical scheme representing the key events taking place in the physicochemical time evolution of our system is summarized in *SI Appendix*, Fig. S14. [This is not a unique system and is only one example of a class of systems capable of undergoing PISA (24, 34, 41, 44, 45)].

By using a protocol of filtrations combined with DLS, NMR, TEM, and optical microscopy of the PISA solution containing the developing vesicles, and monitoring the number of vesicles in filtrate and retentate, we determine and quantify the growth patterns of the populations of vesicles. They follow nonlinear hump-and-trough patterns whose analysis indicates that parent vesicles produced offspring vesicles which, in turn, also reproduced. Our findings are consistent with the congestion of the vesicle membrane by partially reacted and/or degraded shorter, but living, amphiphiles originally in their lumen and which due to chemiosmotic forces and the presence of defects in the membrane, are squeezed out of the vesicle into their heterotrophic environment.

The above scenario is consistent with interpreting the squeezed out, partly polymerized (with a DP below the theoretical value for which the initial blend composition was calculated) and chemically living amphiphiles as chemical spores from which the generational progeny emerges. The simple (conformational) information that gets transferred by these spores is in the structure and length of their partially (and internally modified) reacted amphiphiles whose properties depend on the history of the mother. Their polydispersity index can differ from one generation to the next which provides a primitive and remarkably simple mechanism of heritable variation.

We note that none of the above processes are per se autocatalytic at the chemical level and therefore conclude that the vesicle reproduction processes on which we report here do not require an autocatalytic chemistry. Our process shares some features with what has been discussed by other authors (7, 8, 17, 46), on micellar/vesicular autocatalytic reproduction and recently extended to oscillating vesicles (47) by the Fletcher group. However, our class of autopoietic systems are not chemically autocatalytic but simpler than the above and, due to the lack of initial complexity, are both easier to implement and more probable to occur spontaneously than other experimentally known forms of reproduction, including the system of Sugawara et al. (48) labeled as autoinductive in ref. 49. In our work, it is the rate of polymerization, the value of the CMC and the continuation in the heterotrophic medium of the RAFT polymerization reaction [at different rates inside and outside of the parent vesicle (36)] combined with the imperfect uniformity of the partially reacted internal amphiphiles creeping into the membrane and then getting squeezed out of it, plus the chain length dependence of the CMC and the dynamics of the micelle/vesicle phases (30, 50) together with their network of feedbacks that lead to their self-reproduction.

Finally, we remark that systems physically and chemically analogous to the system described here have recently (41) been shown to display competitive exclusion (4, 51) and therefore Darwinian selection by the struggle for life that goes together with competitive exclusion.

The results presented here have significant implications for chemistry-based abiotic life-like synthetic artificial life and offer a path for developing a broad class of abiotic, life-like systems, for functional materials research, for the applications of vesicles in drug delivery, the study of protocells and their evolution, the origins of life and, finally, to assess the potential for the presence and detection of generalized life (10) on other planetary systems.

## Materials and Methods

In the *SI Appendix* for this paper, we present details of the Synthesis of polymer vesicles, followed by the description of the population growth experiments that we conducted, Data treatment for the acquired light microscope images using ImageJ. Then we discuss the application of Dynamic Light Scattering (DLS), Transmission Electron Microscopy (TEM), NMR, Scanning Electron microscopy (SEM), and Atomic Force Microscopy (AFM) procedures and their details. This is followed by a description and discussion of the Control Experiments for Population Growth Mechanism and Details of the Curve Fitting Procedure. All of which is followed by experiments showing growth curves as functions of changing light irradiation parameters and histograms of the time dependence of the size distributions in the growth experiments. Finally, we offer a Schematic Diagram of the various steps, processes, and major events that take place during the dynamical evolution of our system as a means to orient the reader.

## Supplementary Material

Appendix 01 (PDF)

Dataset S01 (XLSX)

## Data Availability

All study data are included in the article and/or supporting information.

## References

[1] M. Eigen, “What will endure of 20th century biology?” in What Is Life? The Next Fifty Years, M. Murphy, L. A. J. O’Neill, Eds. (Cambridge University Press, New York, 1995).

[2] A. P. Muñuzuri, J. Pérez-Mercader, Unified representation of Life’s basic properties by a 3-species stochastic cubic autocatalytic reaction-diffusion system of equations. Phys. Life Rev. **41**, 64–83 (2022). 35594602 10.1016/j.plrev.2022.03.003

[3] P. Nurse, What Is Life?: Five Great Ideas in Biology (W. W Norton and Co., 2021).

[4] E. Mayr, What Evolution Is (Basic Books, 2001).

[5] A. Murray, T. Hunt, The Cell Cycle. An Introduction (Oxford University Press, New York and Oxford, 1993).

[6] R. Virchow, Die multiloculäire, ulcerirende Echinokokkengeschwulst der Leber. Verhandlungen der Physicalisch-Medicinischen Gesselschaft 84–95 (1855).

[7] P. A. Bachmann, P. L. Luisi, J. Lang, Autocatalytic self-replicating micelles as models for prebiotic structures. Nature **357**, 57–59 (1992).

[8] P. L. Luisi, “Self-reproduction of micelles and vesicles: Models for the mechanisms of life from the perspective of compartmented chemistry” in Advances in Chemical Physics, I. Prigogine, S. A. Rice, Eds. (John Wiliey and Sons Inc, New York, 1996), **vol. XCII**, pp. 425–438.

[9] N. J. Gaut, K. P. Adamala, Reconstituting natural cell elements in synthetic cells. Adv. Biol. **5**, 2000188 (2021), 10.1002/adbi.202000188. 33729692

[10] J. D. Bernal, The Origin of Life (World, 1967).

[11] I. Gözen , Protocells: Milestones and recent advances. Small **18**, 2106624 (2022). 10.1002/smll.202106624 35322554

[12] L. E. Orgel, Self-organizing biochemical cycles. Proc. Natl. Acad. Sci. U.S.A. **97**, 12503–12507 (2000). 11058157 10.1073/pnas.220406697 PMC18793

[13] R. Shapiro, A simpler origin for life. Sci. Am. **296**, 46–53 (2007). 10.1038/scientificamerican0607-46 17663224

[14] J. D. Bernal, “The problem of stages in biopoiesis” in Aspects of the Origin of Life, M. Florkin, Ed. (Pergamon Press Inc., New York, 1960).

[15] R. M. Hazen, Genesis: The Scientific Quest for Life’s Origin (Joseph Henry Press, 2005).

[16] J. Pérez-Mercader, “Making biochemistry-free (generalized) life in a test tube” in The First Steps of Life, E. di Mauro, Ed. (ISTE and J. Wiley, New Jersey, 2024), pp. 135–162, 10.1002/9781394264155.ch7.

[17] L. D. Hurst, R. Dawkins, Life in a test tube. Nature **357**, 198–199 (1992). 1375346 10.1038/357198a0

[18] S. M. Morrow, I. Colomer, S. P. Fletcher, A chemically fuelled self-replicator. Nat. Commun. **10**, 1011 (2019), 10.1038/s41467-019-08885-9. 30824804 PMC6397266

[19] A. N. Albertsen, J. K. Szymański, J. Pérez-Mercader, Emergent properties of giant vesicles formed by a polymerization-induced self-assembly (PISA) reaction. Sci. Rep. **7**, 41534 (2017). 28128307 10.1038/srep41534 PMC5270245

[20] J. Flogeras, Mimicking living systems using polymer chemistry. Advanced Science News, 2018. https://www.advancedsciencenews.com/mimicking-living-systems-using-polymer-chemistry. Accessed 5 May 2025.

[21] S. J. Gould, Life on mars? So what? *New York Times*, 11 August 1996. Opinion page, Section 4, Page 13. https://www.nytimes.com/1996/08/11/opinion/life-on-mars-so-what.html/. Accessed 5 May 2025.

[22] J. Pérez-Mercader, De novo laboratory synthesis of life mimics without biochemistry. Artif. Life Conf. Proc. **32**, 483–490 (2020), 10.1162/isal_a_00282.

[23] D. Huesmann, Macromolecular rapid communications: Polymerization-induced self-assembly. Advanced Science News, 2019. https://www.advancedsciencenews.com/macromolecular-rapid-communications-polymerization-induced-self-assembly/. Accessed 5 May 2025.

[24] Q. Gu, H. Li, E. J. Cornel, J. Du, New driving forces and recent advances in polymerization-induced self-assembly. Cell Rep. Phys. Sci. **4**, 101495 (2023), 10.1016/j.xcrp.2023.101495.

[25] N. J. W. Penfold, J. Yeow, C. Boyer, S. P. Armes, Emerging trends in polymerization-induced self-assembly. ACS Macro Lett. **8**, 1029–1054 (2019), 10.1021/acsmacrolett.9b00464. 35619484

[26] B. Charleux, G. Delaittre, J. Rieger, F. D’Agosto, Polymerization-induced self-assembly: From soluble macromolecules to block copolymer nano-objects in one step. Macromolecules **45**, 6753–6765 (2012).

[27] G. Cheng, J. Pérez-Mercader, Polymerization-induced self-assembly for artificial biology: Opportunities and challenges. Macromol. Rapid Commun. **40**, 1800513 (2019). 10.1002/marc.201800513 30216588

[28] S. D. P. Fielden, M. J. Derry, A. J. Miller, P. D. Topham, R. K. O’Reilly, Triggered polymersome fusion. J. Am. Chem. Soc. **145**, 5824–5833 (2023). 36877655 10.1021/jacs.2c13049 PMC10021019

[29] S. D. P. Fielden, Kinetically controlled and nonequilibrium assembly of block copolymers in solution. J. Am. Chem. Soc. **146**, 18781–18796 (2024), 10.1021/jacs.4c03314. 38967256 PMC11258791

[30] J. N. Israelachvili, Intermolecular and Surface Forces (Elsevier Science, 2011).

[31] S. Pearce, J. Perez-Mercader, Chemoadaptive polymeric assemblies by integrated chemical feedback in self-assembled synthetic protocells. ACS Cent. Sci. **7**, 1543–1550 (2021), 10.1021/acscentsci.1c00681. 34584956 PMC8461774

[32] S. Pearce, C. Lin, J. Pérez-Mercader, Adaptive and dissipative hierarchical population crowding of synthetic protocells through Click-PISA under gradient energy inputs. ACS Nano Lett. **24**, 2457–2464, 10.1021/acs.nanolett.3c04035. PMC10906081 38373157

[33] M. Hartlieb, Photo-iniferter RAFT polymerization. Macromol. Rapid Commun. **43**, 2100514 (2022), 10.1002/marc.202100514. 34750911

[34] S. Pearce, J. Perez-Mercader, PISA: Construction of self-organized and self-assembled functional vesicular structures. Polym. Chem. **12**, 29–49 (2021), 10.6561039/D0PY00564A.

[35] M. Lansalot, J. Rieger, F. d’Agosto, “Polymerization-induced self-assembly: The contribution of controlled radical polymerization to the formation of self-stabilized polymer particles of various morphologies”, Chapter 2 in Macromolecular Self-Assembly, L. Billon, O. Borisov, Eds. (John Wiley and Sons, New Jersey, 2016), pp. 33–82.

[36] R. Takahashi , Unraveling the kinetics of the structural development during polymerization-induced self-assembly: Decoupling the polymerization and the micelle structure. Polym. Chem. **11**, 1514–1524 (2020), 10.1039/C9PY01810G.

[37] G. Odian, Principles of Polymerization (Welly Interscience, 2004), 10.1002/047147875X.index.

[38] S. Shanmugam, J. Xu, C. Boyer, Exploiting metalloporphyrins for selective living radical polymerization tunable over visible wavelengths. J. Am. Chem. Soc. **137**, 9174–9185 (2015). 26167724 10.1021/jacs.5b05274

[39] N. J. Warren , Testing the vesicular morphology to destruction: Birth and death of diblock copolymer vesicles prepared via polymerization-induced self-assembly. J. Am. Chem. Soc. **137**, 1929–1937 (2015), 10.1021/ja511423m. 25526525 PMC4333598

[40] C. Lin, S. K. Katla, J. Pérez-Mercader, Photochemically induced cyclic morphological dynamics via degradation of autonomously produced, self-assembled polymer vesicles. Commun. Chem. **4**, 25 (2021), 10.1038/s42004-021-00464-8. 36697697 PMC9814595

[41] S. K. Katla, C. Lin, J. Pérez-Mercader, Competitive exclusion principle among synthetic non-biochemical protocells. Cell Rep. Phys. Sci. **4**, 101359 (2023).

[42] C. Mayer, D. Lancet, O. Markovitch, The GARD prebiotic reproduction model described in order and complexity. Life **14**, 288 (2024). 38541614 10.3390/life14030288 PMC10971654

[43] D. H. Meadows, D. L. Meadows, J. Randers, W. Behrens III, The Limits to Growth (A Potomac Associate Book, Universe Books, New York, 1972).

[44] J. Wan, B. Fan, S. H. Thang, RAFT-mediated polymerization-induced self-assembly (RAFT-PISA): Current status and future directions. Chem. Sci. **13**, 4192–4224 (2022), 10.1039/d2sc00762b. 35509470 PMC9006902

[45] J. C. Foster , 100th anniversary of macromolecular science viewpoint: The role of hydrophobicity in polymer phenomena. ACS Macro Lett. **9**, 1700–1707 (2020), 10.1021/acsmacrolett.0c00645. 33299653 PMC7717397

[46] M. G. Howlett, S. P. Fletcher, From autocatalysis to survival of the fittest in self-reproducing lipid systems. Nat. Rev. Chem. **7**, 673–691 (2023), 10.1038/s41570-686023-00524-8. 37612460

[47] Z. Zhang, M. G. Howlett, E. Silvester, P. Kukura, S. P. Fletcher, A chemical reaction network drives complex population dynamics in oscillating self-reproducing vesicles. J. Am. Chem. Soc. **146**, 18262–18269 (2024). 38917079 10.1021/jacs.4c00860 PMC11240260

[48] K. Takakura, T. Toyota, T. Sugawara, A novel system of self-reproducing giant vesicles. J. Am. Chem. Soc. **125**, 8134–8140 (2003). 12837083 10.1021/ja029379a

[49] D. G. Blackmond, An examination of the role of autocatalytic cycles in the chemistry of proposed primordial reactions. Angew. Chem. Int. Ed. Engl. **48**, 386–390 (2009). 19053125 10.1002/anie.200804565

[50] A. Sánchez-Iglesias , Hydrophobic interactions modulate self-assembly of nanoparticles. ACS Nano **6**, 11059–11065 (2012). 23186074 10.1021/nn3047605

[51] E. Mayr, This is Biology. The Science of the Living World (Harvard University Press, Cambridge, MA, 1997).

