# Supplementary material for: Self-reproduction as an autonomous process of growth and reorganization in fully abiotic, artificial and synthetic cells
Source: Proc Natl Acad Sci U S A. 2025 May 27;122(22):e2412514122. doi: 10.1073/pnas.2412514122 (PMC12146737; doi:10.1073/pnas.2412514122)
Supplement: Supplementary file 1 — Appendix 01 (PDF) [file pnas.2412514122.sapp.pdf]

Supplementary Information

Self-reproduction as an Autonomous Process of Growth and Reorganization in Fully Abiotic, Artificial and Synthetic Cells

Sai Krishna Katla,[†] Chenyu Lin,[†] Juan Pérez-Mercader [†§*]

[†]Department of Earth and Planetary Sciences, Harvard University, Cambridge, Massachusetts 02138-1204, United States

[§]The Santa Fe Institute, Santa Fe, New Mexico 87501, United States

Materials & Methods

Synthesis of polymer vesicles: The active and functional polymer vesicle population was created using light-mediated controlled radical polymerization using a photo-iniferter. Photo-iniferters are a type of Reversible Addition−Fragmentation chain-Transfer (RAFT) agents. They can absorb photon energy and direct it to initiate and control radical polymerization for extending polymer chains, eliminating the need for photocatalysts, **Figure S1**. In a typical synthesis, 9.8 mg of polyethyleneglycol-4-cyano-4-[(dodecylsulfanylthiocarbonyl)sulfanyl] pentanoic acid (photo-iniferter, M_n = 1400) was mixed with 147 μL 2-hydroxypropyl methacrylate (monomer, M_n = 144.17) in 1.5 mL of water in a 1-dram vial. The vial is vortexed for 5 min and closed with a rubber stopper. Using two syringe needles (for inlet and outlet of gas) through the rubber stopper, the mixture in the vial was bubbled with N_2 gas for 10 min to minimize dissolved oxygen. The vial was then placed in the center of a custom-built photoreactor, **Figure S2**, and irradiated for 90 min. The temperature was maintained at 33 ^{o}C during the reaction. The custom photoreactor was created with a strip of 45 green LEDs (λ = 523 nm; single LED power = 2.97 mW) circularly wound around the outside surface of a 100 mL glass beaker. The reactor was wrapped with aluminum foil to avoid stray light entering the container during the reaction.Light irradiation was stopped after 90 min and the thick cloudy white polymer product that was formed was diluted to 0.2% v/v in water (HPLC grade) to make stable colloidal solutions for further characterizations and for use in the population growth experiments.

Population growth experiments: 100 uL of the 0.2% diluted polymer vesicle suspension was added to a 1 mL Eppendorf tube, followed by a 10% v/v addition of ZnTPP (400 μM in DMSO). After gently swirling the mixture, it was transferred to a frame-sealed slide to prepare the microscopic specimen. The specimen then underwent a 12-hour irradiation period under the microscope (using EVOS Auto FL 2 Cell Imaging Systems from Invitrogen mounted on a Thorlabs ScienceDesk™ Workstation with a 2.5' by 4.0' Breadboard equipped with Active, Self-levelling Vibration Isolation) with the following irradiation pattern: green light (530 nm) with a 1 s pulse duration, white light with a 0.5 ms pulse duration, and a 10 s pulse period. The resulting time-lapse image stacks were analyzed to determine the number and average vesicle-size of the polymer vesicle population.

Data treatment for Microscopic Image Stack: The analysis of resulting time-lapse images was processed for evaluating the temporal changes in the number and the average size of the population using ImageJ software. The following functions of ImageJ were applied to the opened image stacks prior to the number and the size analysis:

(a) Subtract Background

(b) Bandpass Filter

(c) Convert to Mask (threshold)

(d) Fill Holes

(e) Watershed

The processed image stacks were then analyzed using the (f) "Analyze Particles" function of ImageJ to obtain the number and the average size values as a function of time for the

subsequent plotting process. The average size (or average diameter) reported at each time value was obtained using the ImageJ "Analyze Particles" function to analyze each EVOS microscopic movie frame captured by the microscope camera. This function looks at the frame and identifies the objects (vesicles) using functions (a) to (e) above, providing the number of pixels in each object. Function "Analyze Particles" applies the calibration value in units of pixel/μm for the objective used in the analysis obtained by using a Thermo-Fisher AMEP-4720[02] EVOS calibration slide, which for the 10X turns out to be 1.4083 pixel per μm. The average diameter in μm obtained using this method is the value quoted in column 3 of the Excel sheet corresponding to each of the three experiments reported in the experimental data for this paper.

Dynamic Light Scattering (DLS) procedure: 1 mL of 0.2% diluted polymer vesicle suspension was transferred into a capped disposable cuvette (Malvern, product number: DT0012). The cuvette was then placed in the temperature-controlled chamber (at 19°C) of a DLS instrument (Zetasizer Ultra, Malvern) for measurement of hydrodynamic diameter and particle size distribution of as-synthesized polymer vesicles.

Transmission Electron Microscopy (TEM) Procedure: a small aliquot of the synthesized polymer vesicles was placed on a 400-mesh copper grid (PELCO from Ted Pella Inc.). The polymer vesicles were then allowed to adhere to the grid's surface for a period of two minutes. Next, a piece of filter paper was used to blot the grid, effectively removing any excess liquid. The grid was left to dry overnight in a dark setting. Finally, a Hitachi HT7800 electron microscope (operating at 80 kV) was used to image the polymer vesicles. The grid was placed inside the instrument and images were acquired in low and high magnification

modes. To avoid electron beam damage to the polymer vesicles, low voltage (80 kV) was used for the microscopy imaging.

Proton Nuclear Magnetic Resonance (1H-NMR) Procedure: First, 50 uL of the suspension was taken that has the synthesized polymer vesicles and added to an Eppendorf tube that already contained 550 uL of methanol-D4. Next, the mixture was vortexed for 5 minutes and then moved to an NMR tube. A 500 MHz Varian Unity/Inova spectrometer was then used to detect the structure of the synthesized amphiphiles in the prepared NMR sample.

Scanning electron microscopy (SEM): A small aliquot of the as-synthesized polymer vesicle suspension was dropped on a piece of silicon wafer (1 cm x 1 cm) and dried overnight in a dark environment. The silicon wafer was then adhered to the top surface of a stub using carbon tape followed by sputter coating of Pd metal using EMS 300T D Dual Head Sputter Coater. The prepared sample was then analyzed using SEM instrument, FESEM Supra55VP, operating at 3 kV.

Atomic Force Microscopy (AFM): The topography of the polymer vesicles along with its Young's modulus and adhesion force was analyzed using a liquid-mode atomic force microscope. For this, 100 μL of the polymer vesicles solution was placed on a glass slide and the sample was placed on a Zeiss microscope equipped with Bruker's JPK BioNano liquid-mode AFM. The polymer vesicles were then imaged to analyze the topography within the liquid using tapping mode AFM and the Young's modulus and adhesion force were analyzed on a single vesicle using contact-mode. The polymer vesicles were then irradiated with blue light from the microscope for 30 min and the effect of irradiation on the size and shape of the vesicles was also studied.

Control experiments for population growth mechanism: For the polymer vesicle population without ZnTPP photocatalyst, a 0.2% diluted suspension of as-synthesized polymer vesicles was filtered using 0.22 μm Polytetrafluoroethylene (PTFE) filter. This process separated the suspension into two parts: the retentate which contains the polymer vesicles and the filtrate which contains the unpacked amphiphiles, unreacted HPMA, and small micelles. The polymer vesicles were then resuspended in water (18 MΩ) containing HPMA at a concentration equivalent to that in the 0.2% diluted suspension. The resuspended polymer vesicles and the filtered amphiphile filtrate were respectively transferred to frame-sealed chambers on a slide for microscope specimen preparation. The specimens underwent irradiation using an EVOS microscope, with two light pulses (for every 10 s pulse period, the specimen was under irradiation of green light with 1 s as pulse duration followed by white light with 0.5 ms as pulse duration and stayed in darkness for the rest of the period) applied.

For the polymer vesicle population with a 40 μM ZnTPP photocatalyst 10% v/v ZnTPP (400 μM in DMSO) was firstly added to the 0.2% diluted as-synthesized polymer vesicle suspension. The ZnTPP loaded suspension then underwent the same procedure as that without ZnTPP (please refer to the above paragraph).

Curve fitting procedure: The presented patterns of the count and the size in **Figure 3** of the main text are the averaged value of the individual observations for the count and size of the vesicles present in three repetitions of the experiment. The clock was always started at the initiation of each experiment. The data points obtained in each experiment were firstly obtained by analysis of objects present on each time-lapse image every 50 s using ImageJ. The resulting count of the number of vesicles and their individual size observed in each of

these three separate experiments were then averaged at each moment of elapsed time since the beginning of the experiment to give the data points and their standard errors shown in **Figure 3** (to avoid visual clutter only 100 error bars for each curve are displayed in the figure).

The curve fitting for the mean count in Figure 3 was performed using the BiDoseResp equation, see Beckon et al. [1],

$$y = A1 + (A2 - A1)\left[\frac{p}{1 + (x_{01}/10^{x})^{h1}} + \frac{1-p}{1 + (x_{02}/10^{x})^{h2}}\right]$$

as a built-in function in the Origin 2023 software package (Origin Lab). The trends for the mean count of the population were divided into three stages based on the behaviors of the first and second derivatives of the BiDoseResp fitting curve (cf. **Figure S11** below). The description of the fitting model parameters and their interpretations can also be found on reference [1] in this SI. This class of models (BiPhasic as they are called in [1]) are models "in which the dependent variable is maximal or minimal at an intermediate value of the independent variable and are known to appear when modelling the growth of reproducing biological populations where there is a reversal of system response between lower and higher doses of some essential system "affecter" that changes in a logarithmic scale". An example of such affecter can be the DP in a polymerization reaction as a function of the concentration or of time. Indeed, although the time-scale in **Figure 3** is linear, instead of using time in the x-axis of this figure, we can think qualitatively of using it as a proxy for the logarithmic representation of the degree of polymerization (DP) of the HPMA monomers into the PHPMA blocks of the amphiphile as a function of time [38] (cf. reference [38] of the main

text, reference [2] in this SI). The discussion in the main text has this notion as its background.

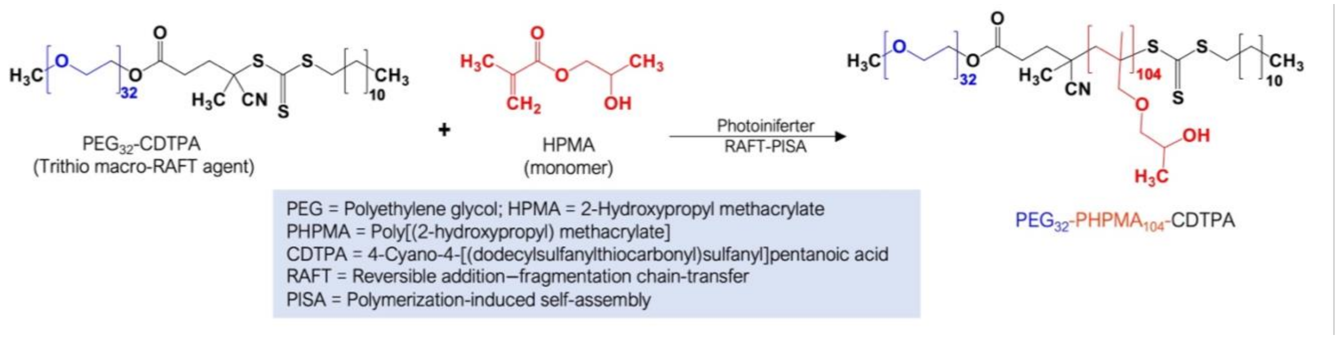

Figure S1. Scheme of the photoiniferter RAFT polymerization reaction for the synthesis of PEG_{32}-$PHPMA_{104}$-CDTPA vesicles.

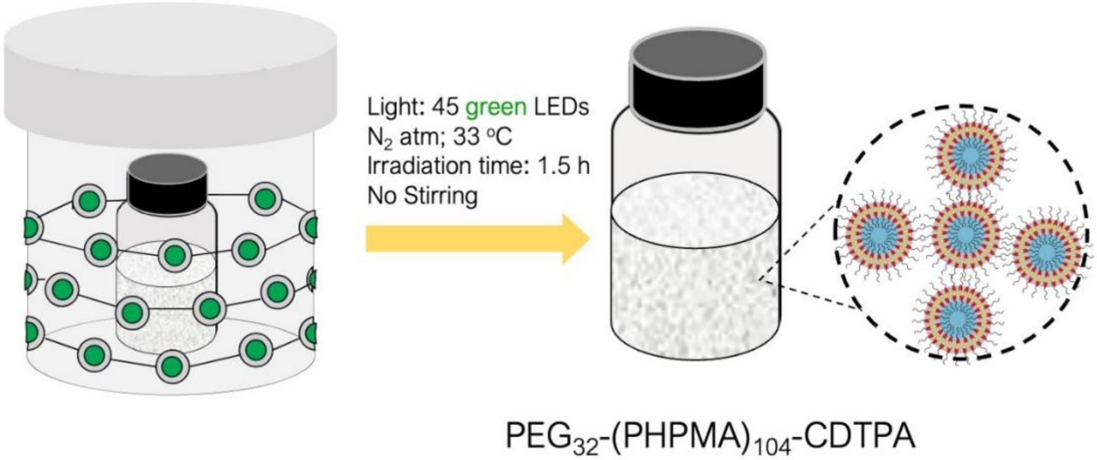

Figure S2. Illustration of photochemical reactor and the reaction conditions.

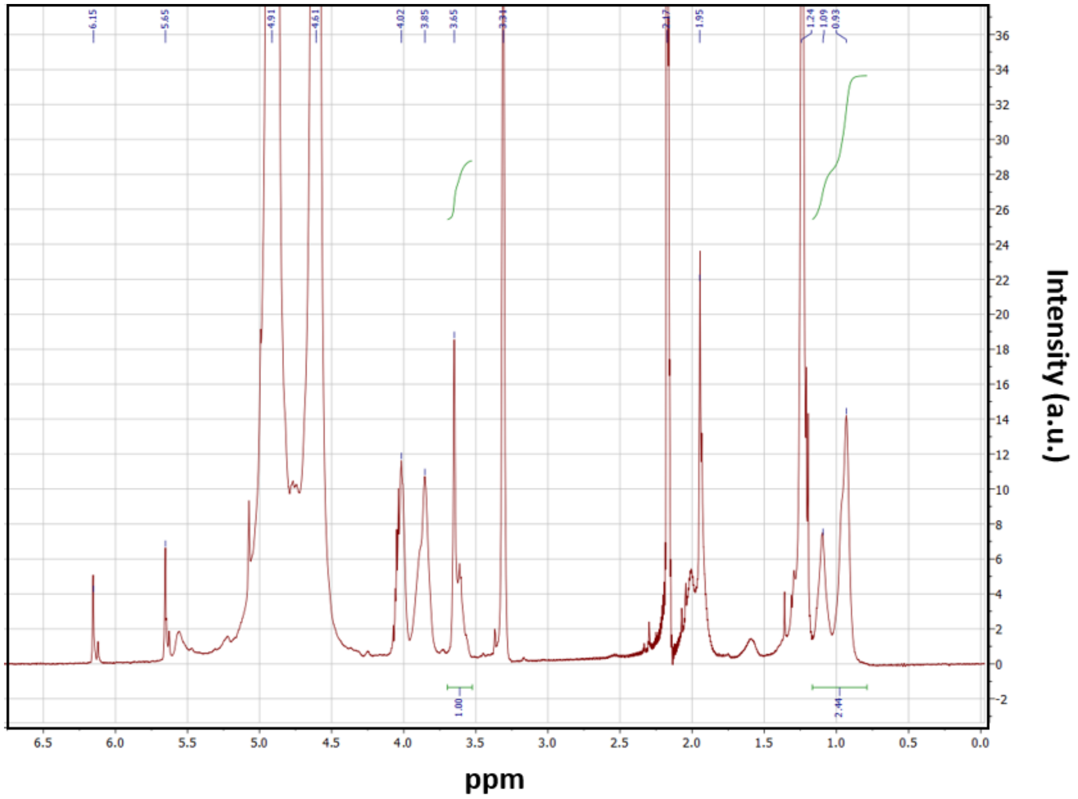

Figure S3. ^{1}H NMR spectrum of the as-synthesized polymer after 90 min. The ^{1}H NMR peaks corresponding to the PHPMA and PEG are at 1 ppm and 3.6 ppm respectively. The PEG peak of the sample before and after polymerization is integrated and compared to calculate the number of PHPMA units or the degree of polymerization.

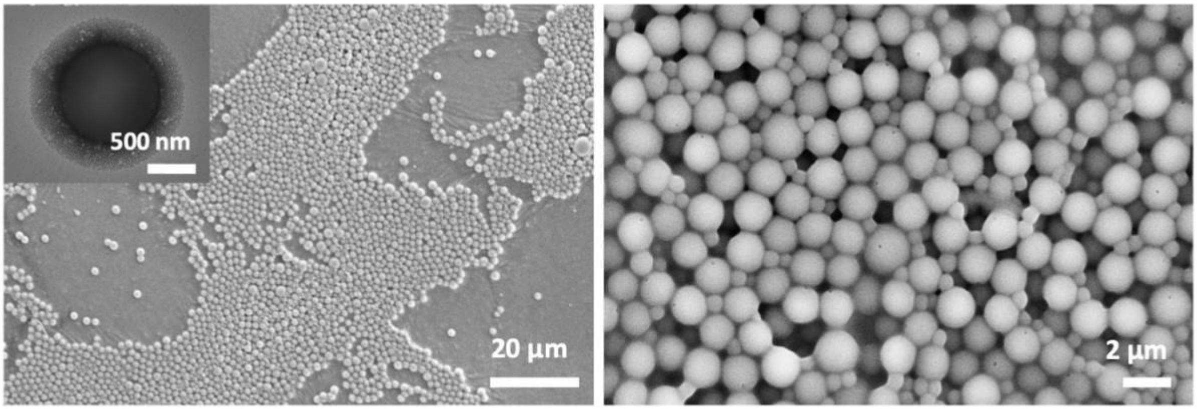

Figure S4. SEM images showing the low magnification and TEM (inset) image showing the hollow core of the vesicles.

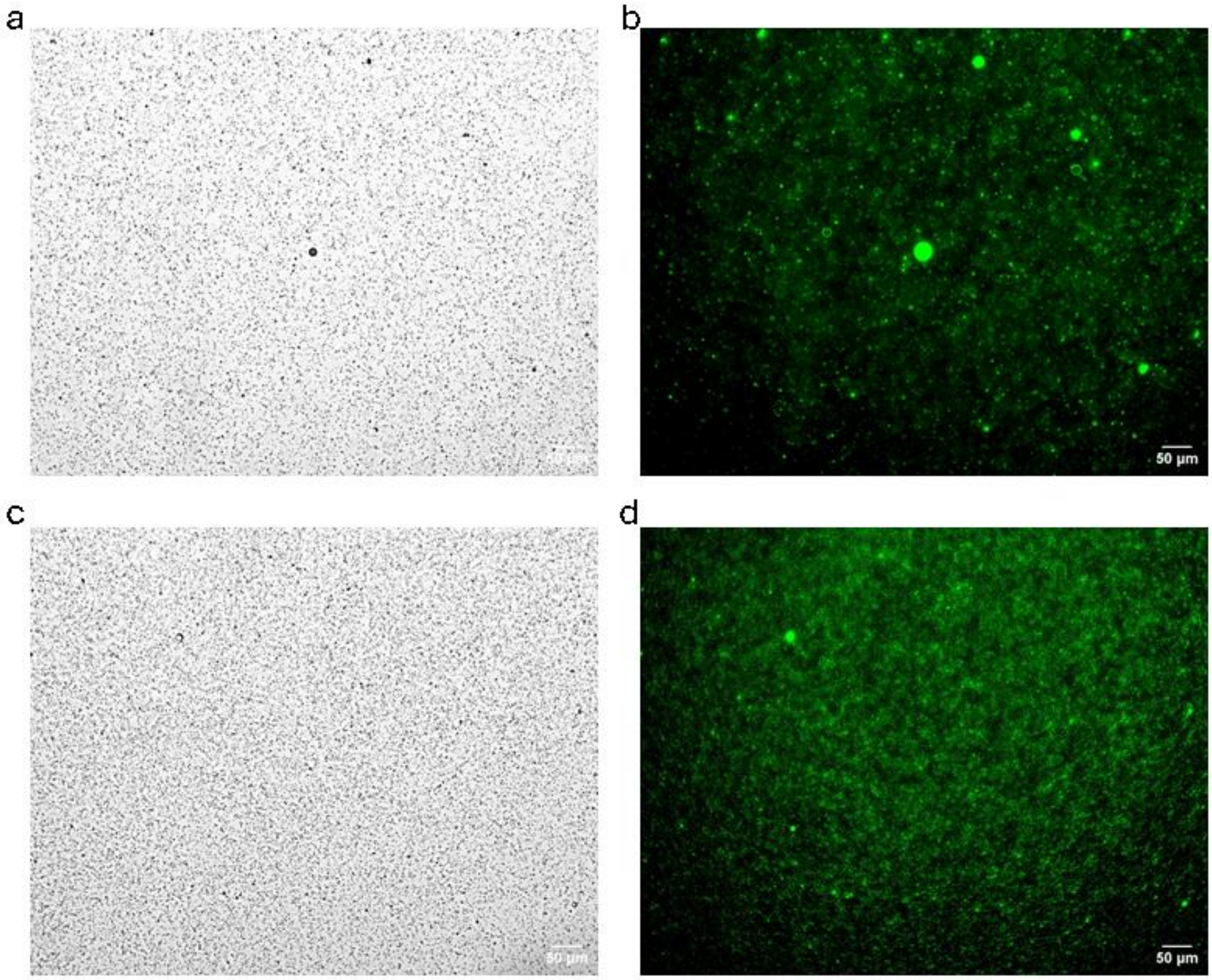

Figure S5. EVOS microscopy for vesicle population before irradiation (**a.** bright field, **b.** fluorescence) and after irradiation (**c.** bright field, **d.** fluorescence)

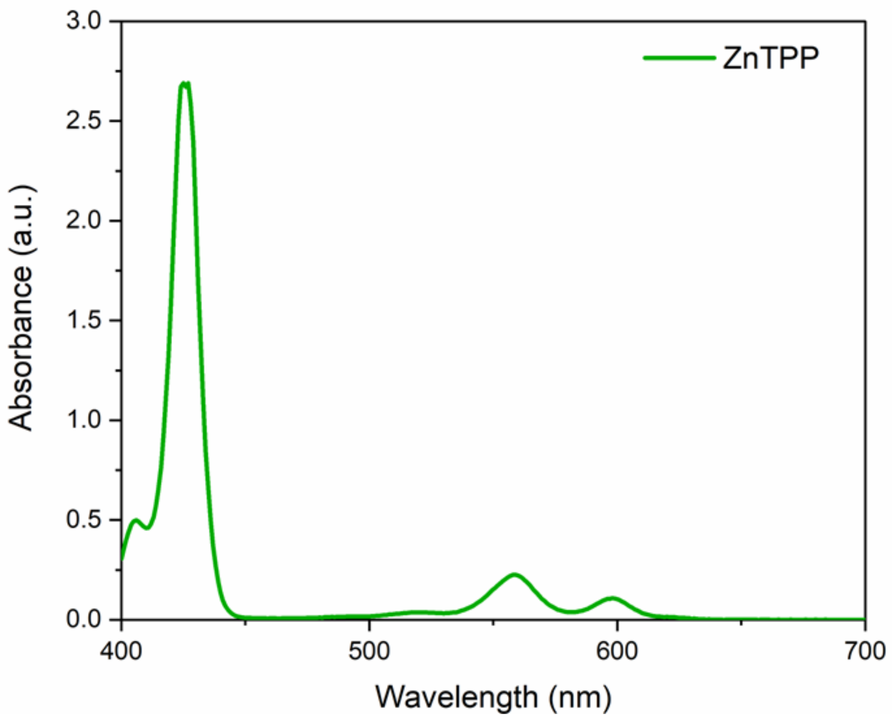

Figure S6. UV-VIS spectroscopy for ZnTPP (0.027 mM in DMSO).

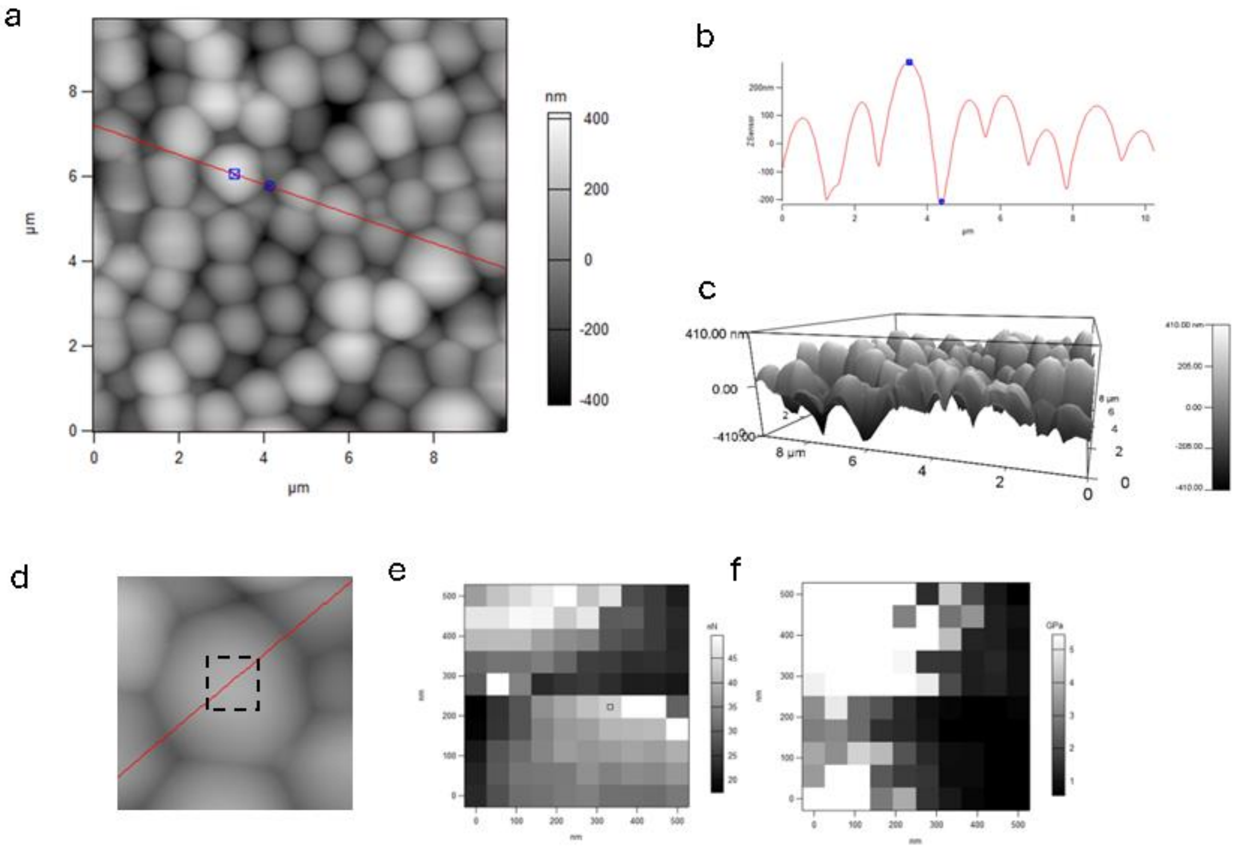

Figure S7. Images acquired using JPK BioNano AFM (Bruker): **a.** AFM image of polymer vesicles, **b.** Depth profile of the polymer vesicles from panel **a**. **c.** The cross-sectional 3-D view of the vesicles. **d.** Single vesicle with highlighted region where adhesion force **e** and Young's modulus **f** were measured using contact-mode AFM.

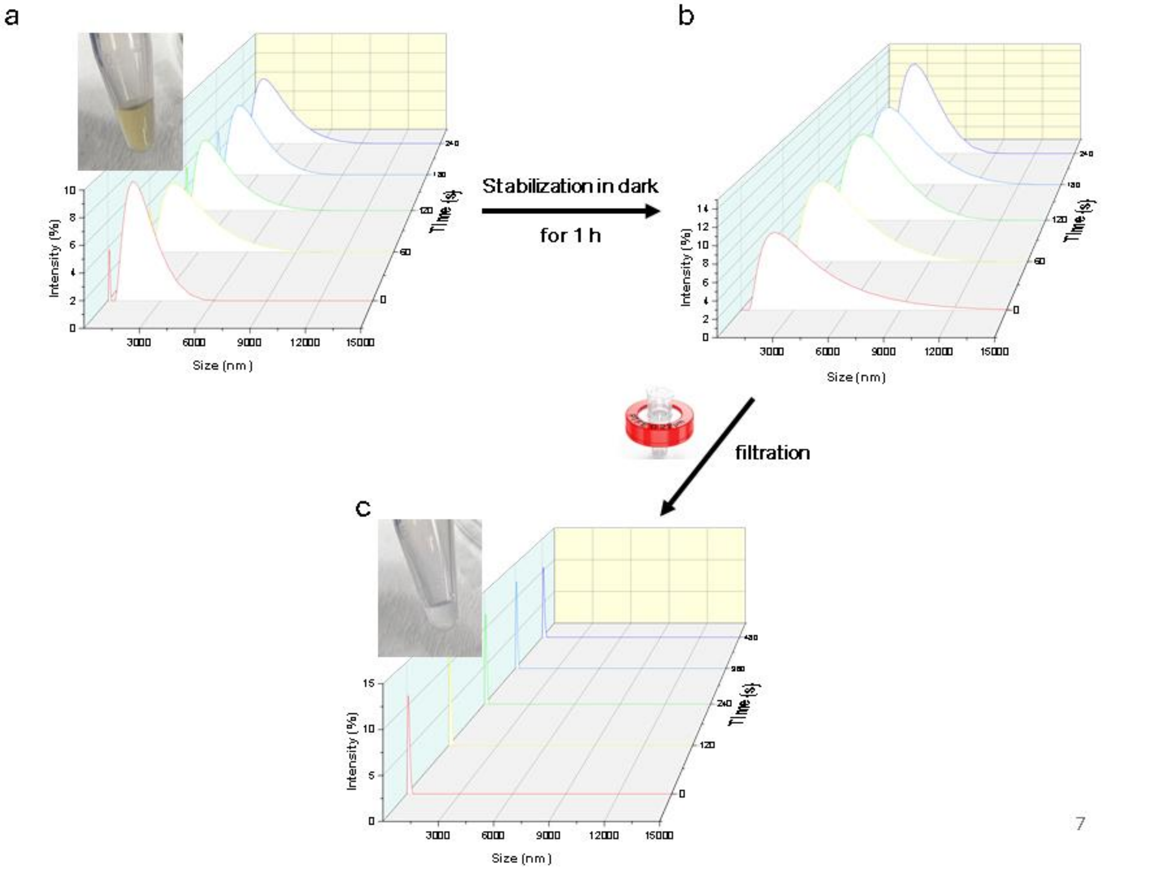

Figure S8. Dynamic light Scattering (DLS) study (capillary) after filtration (0.22 µm filter) **a.** before filtration (Size of peak 1: 1828 ± 136.5 nm; Size of peak 2: 117.8 ± 18.85 nm, **b.** after 1-hour stabilization in dark (Size: 2090 ± 259.2 nm), **c.** After filtration (size: 73.26 ± 1.95 nm).

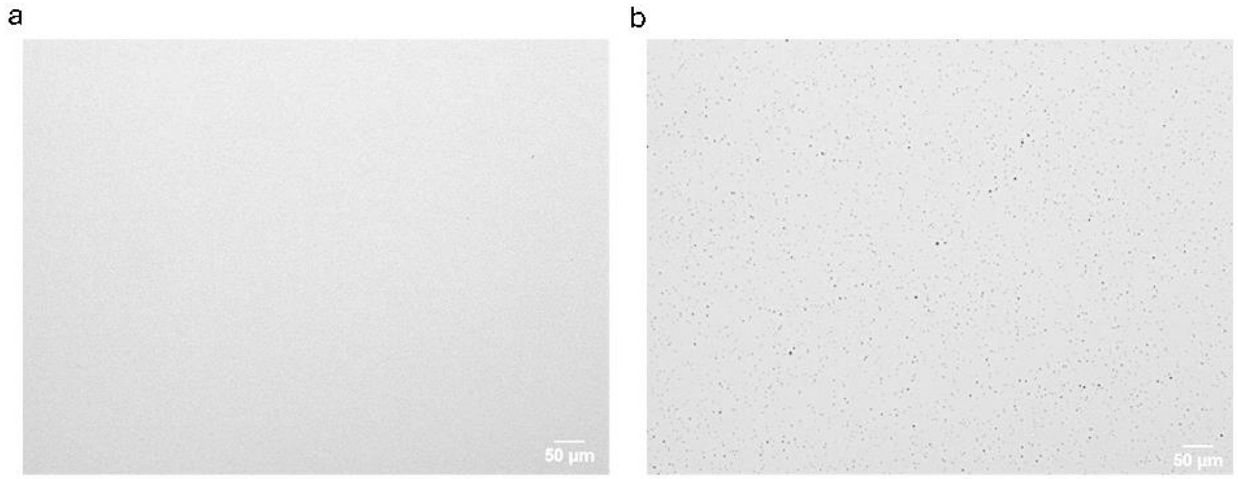

Figure S9. EVOS microscope bright field imaging for PISA undergoing filtration process: **a.** filtrate, **b.** retentate.

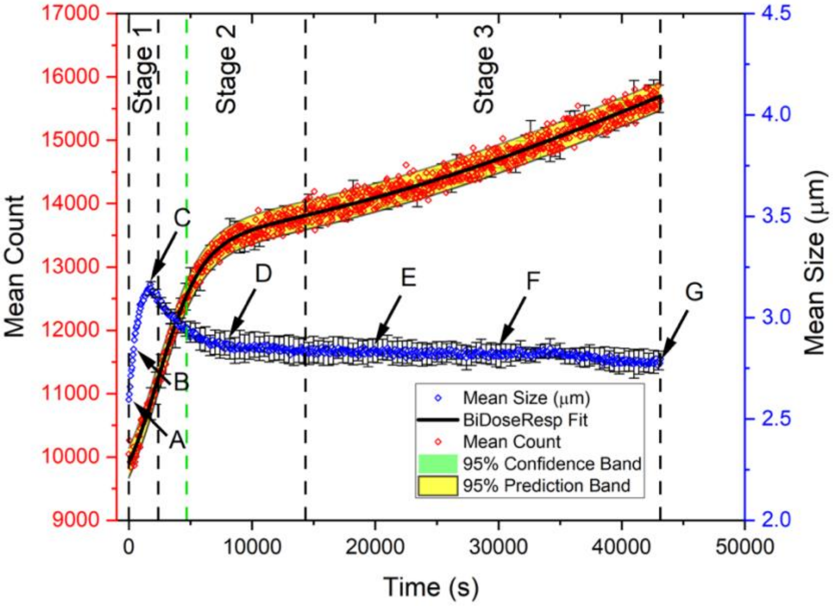

Mean Count of Population	
Model	BiDoseResp
A1	8485 ± 211
A2	18963 ± 1856
LOGx01	2366 ± 130
LOGx02	45413 ± 9454
h1	2.47E-4 ± 1.08E-5
h2	2.26E-5 ± 5.8E-6
p	0.41± 0.09
Reduced Chi-Sqr	13081.26
R-Square (COD)	0.991
Adj. R-Square	0.991

Figure S10. BiDoseResp curve fitting results for the number of objects (red rhombs) and the average size (blue circles) of polymer vesicles (containing ZnTPP molecules) produced as a function of time as the PISA system is irradiated with the green light of our EVOS microscope. The "Mean Count" and "Mean Size" are the value of the population number of vesicles and their diameters in the microscope averaged for each value of the time coordinate for three independent repetitions of our reproduction experiments. The number of images processed for each experiment is 4320 (each of 1.6-1.8 Mb) during a 12 hour experiment, but to avoid clutter we only have plotted in this figure one fifth of them, that is 864 data points per experiment which were then averaged and plotted in this figure. The error bars represent the value of the SDOM (Standard Deviation Of the Mean) for each point; again to avoid clutter we have represented only one error bar for each ten consecutive points. The 95% Confidence Band is about the width of the black line

representing the fit to the mean value of the three experiments. The fit details are in the right hand side panel of this figure, and the parameters correspond to the ones pertaining to the Curve Fitting Procedure of this SI. The Stages in the figure represent regions where the first derivative (corresponding to kinetics) of the population curve changes sign, The broken vertical green line is the inflection point of the first derivative as seen in the second derivative (dynamics). The letters A-G on the Mean Size curve denote the time-stamp values for the histograms in **Figure S13**.

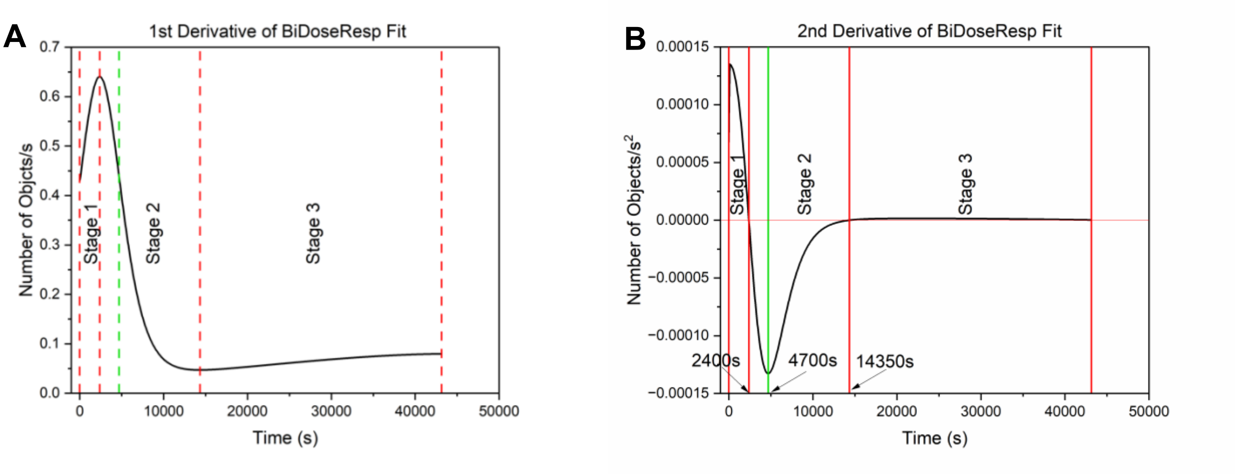

Figure S11. Population growth rate (kinetics) curve and its derivative (dynamics) for the fitted vesicle number curve of Figure 3 (also in Figure S10). These derivatives are obtained numerically from the first and second derivative with respect to time of the fitted curve for the observed population as function of time.

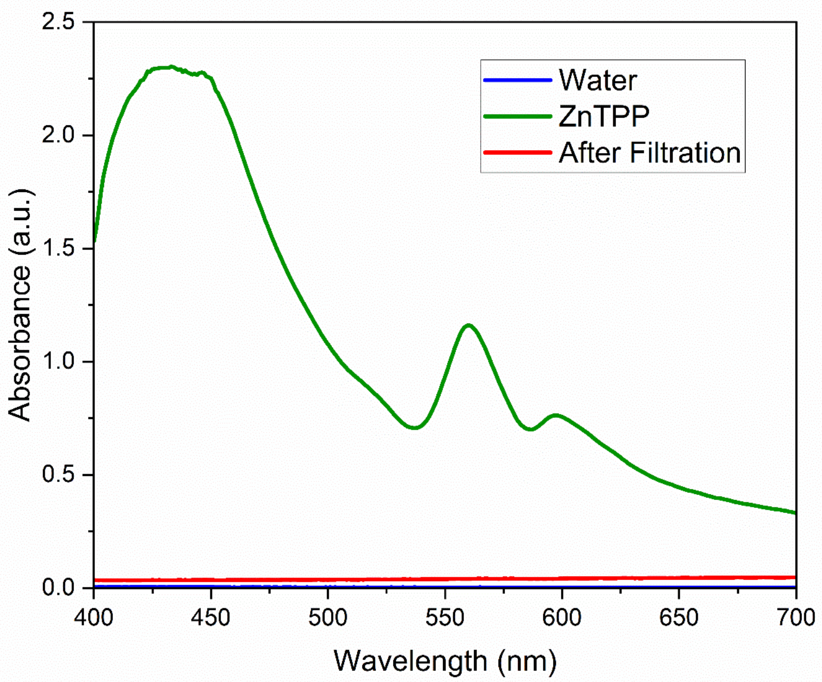

Figure S12. Visible absorption spectra of ZnTPP loaded vesicles before and after filtration. The ZnTPP absorbance is practically zero in the filtered sample.

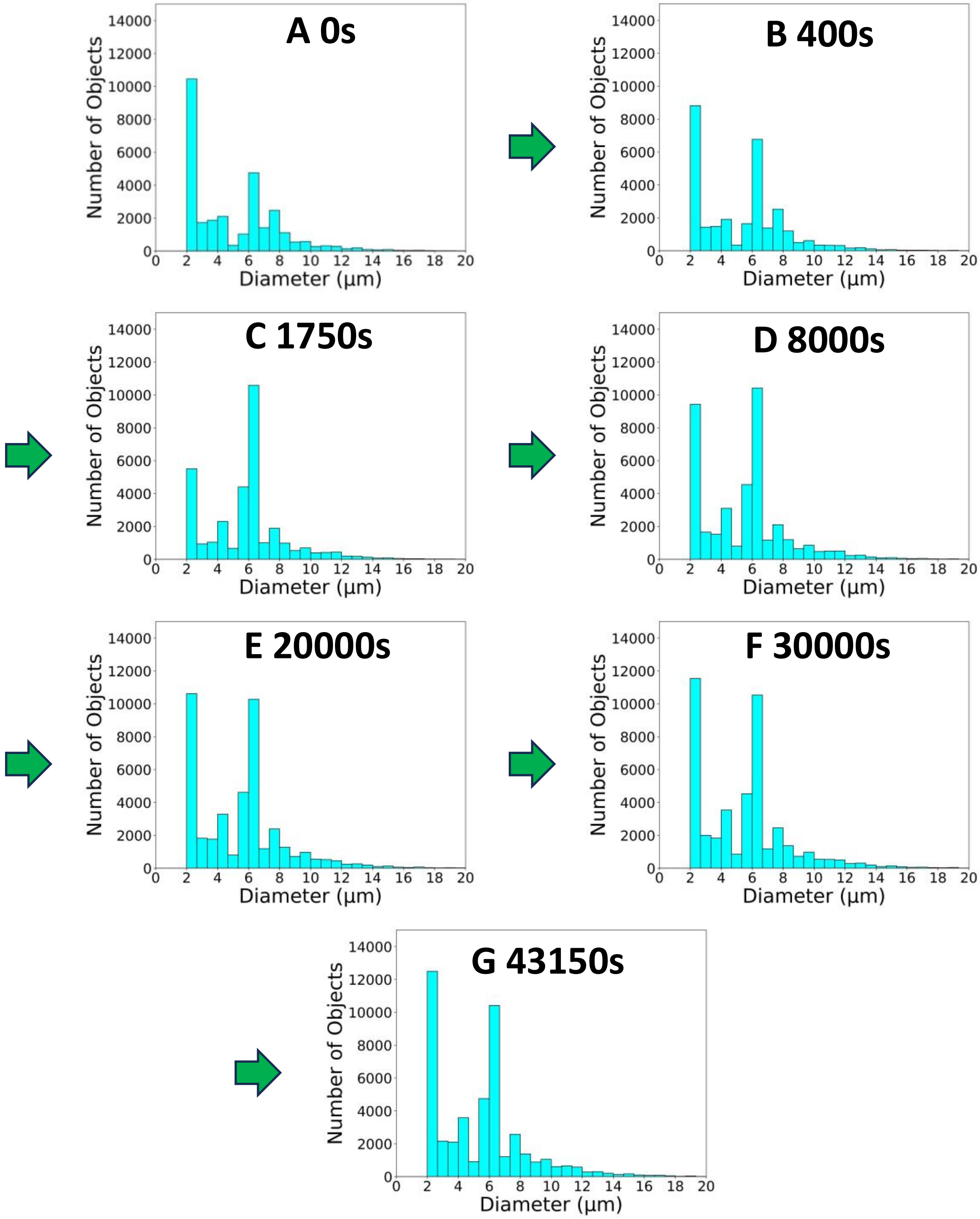

Figure S13. Histograms (bin width: 0.667 µm, total bin range: 0 ~ 20 µm) of size distribution at different time points for the combination of the three repetitions of our reproduction experiments. The time stamp of each histogram corresponds to the time point of the average size used in studying the data represented in Figure 3 of the paper. The time-dependent multimodal distribution and the change in the number of peaks and in their intensity and relative correlations as time elapses are, together with the non-linearity of the population growth curve, signatures of the reproduction process taking place in our system.

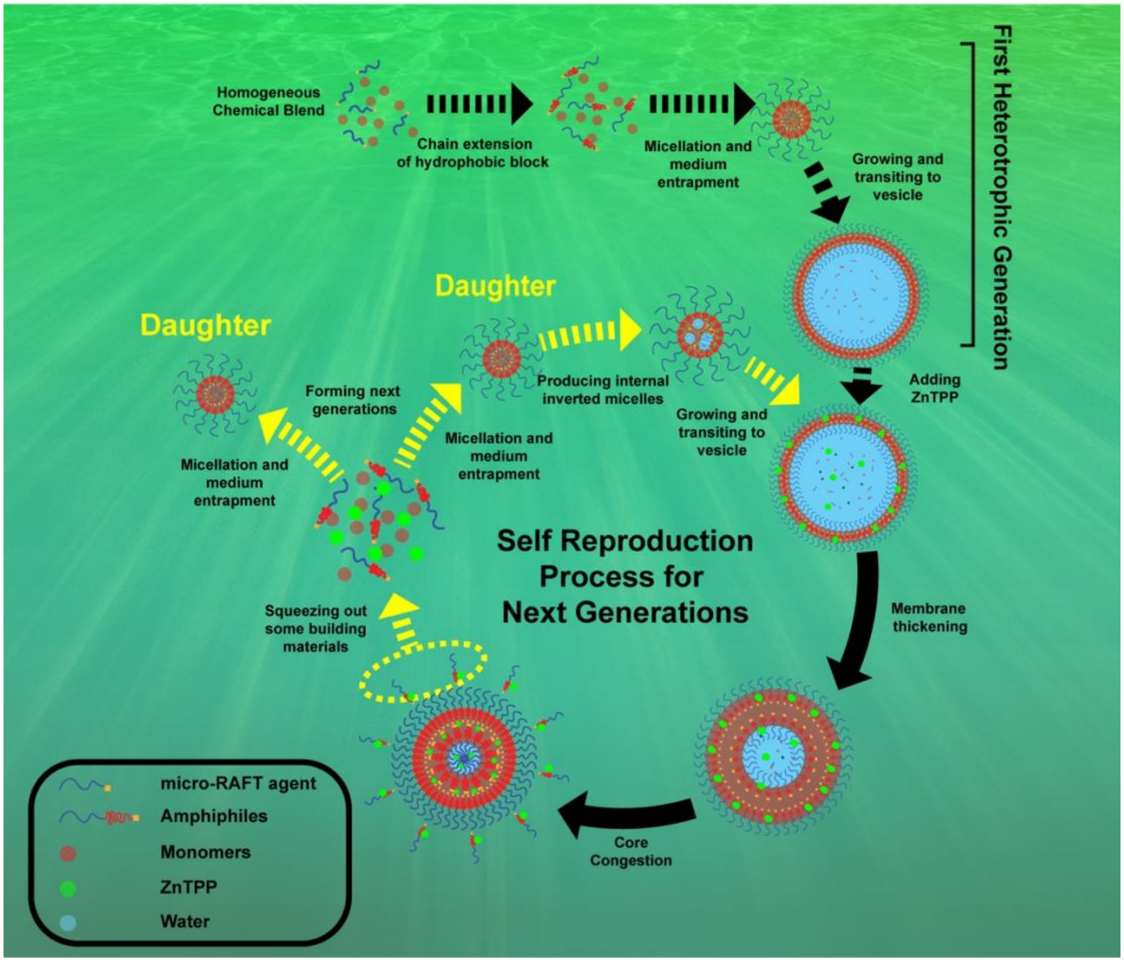

Figure S14 Schematic diagram summarizing the various steps and processes associated with the dynamical course of our system. At the top we have the first (heterotrophic and autopoietic) generation, which once it takes up a catalyst (not a necessary step, but very useful for experimental purposes) develops into a self-reproducing vesicular system. Notice that it contains a number of steps (or physico-chemical phases) that are the result of various molecularly powered feedbacks between polymerization, degradation, chemiosmotic gradients, monomer availability and energy supplied to the system (through pulsed green 530 nm light, represented by the background of the picture). These steps are labelled according to the processes taking place. Inset is a legend with the keys to the various symbols.

References

1. Beckon, W. M., Parkins, C., Maximovich, A. and Beckon, A. V., A general approach to Modelling Biphasic Relationships, *Environ. Sci. Technol.* 2008, *42*, 1308-1314.
2. Warren, N. J. *et al.* Testing the Vesicular Morphology to Destruction: Birth and Death of Diblock Copolymer Vesicles Prepared via Polymerization-Induced Self-Assembly. *J. Am. Chem. Soc.* **137**, 1929–1937 (2015). DOI: 10.1021/ja511423m
